# Multimodality therapy approaches, local and systemic treatment, compared with chemotherapy alone in recurrent glioblastoma

**DOI:** 10.1186/s12885-015-1488-2

**Published:** 2015-06-30

**Authors:** Marta Scorsetti, Pierina Navarria, Federico Pessina, Anna Maria Ascolese, Giuseppe D’Agostino, Stefano Tomatis, Fiorenza De Rose, Elisa Villa, Giulia Maggi, Matteo Simonelli, Elena Clerici, Riccardo Soffietti, Armando Santoro, Luca Cozzi, Lorenzo Bello

**Affiliations:** 1 Radiotherapy and Radiosurgery Department, Humanitas Research Hospital, Humanitas Cancer Center, Istituto Clinico Humanitas, Via Manzoni 56, 20089 Rozzano, Milano Italy; 2 Neuro-oncological Surgery Department, Humanitas Cancer Center and Università degli Studi di Milano, Milan, Italy; 3 Neuroncology Department Le Molinette, Turin, Italy; 4 Oncology and Hematology Department, Humanitas Cancer Center, Milan, Italy

**Keywords:** Glioblastoma, Recurrence, Retreatment

## Abstract

**Background:**

Long-term local control in Glioblastoma is rarely achieved and nearly all patients relapse. In this study we evaluated the clinical effect of different treatment approaches in recurrent patients.

**Methods:**

Forty-three patients, with median age of 51 years were evaluated for salvage treatment: re-resection and/or re-irradiation plus chemotherapy or chemotherapy alone. Response was recorded using the Response Assessment in Neuro-Oncology criteria. Hematologic and non-hematologic toxicities were graded according to Common Terminology Criteria for Adverse Events 4.0. Twenty-one patients underwent chemotherapy combined with local treatment, surgery and/or radiation therapy, and 22 underwent chemotherapy only.

**Results:**

The median follow up was 7 months (range 3–28 months). The 1 and 2-years Progression Free Survival was 65 and 10 % for combined treatment and 22 and 0 % for chemotherapy alone (*p* < 0.01). The 1 and 2-years overall survival was 69 and 29 % for combined and 26 and 0 % for chemotherapy alone (*p* < 0.01). No toxicity greater than grade 2 was recorded.

**Conclusion:**

These data showed that in glioblastoma recurrence the combination of several approaches in a limited group of patients is more effective than a single treatment alone. This stress the importance of multimodality treatment whenever clinically feasible.

## Background

Despite the use of maximal surgical resection, followed by radiotherapy with concomitant and adjuvant temozolomide (TMZ) improved survival in newly diagnosed glioblastoma (GBM), recurrence is still a significant problem affecting more than 90 % of patients with this disease [1]. The median overall survival (OS) is 15–18 months and less than 10 % of patients are still alive at 5 years [2]. Long-term local or regional control is rarely achieved and nearly all patients relapse [3]. To date, several, non-randomized, clinical trials on recurrence are available, with heterogeneous patient cohorts, several treatment approaches, and different endpoints recorded. Different approaches are used including re-resection [4, 5], chemotherapy [6, 7] or re-irradiation [8–11]. Surgery is an effective option only in selected patients with younger age (70 years or less), a small tumor volume (<50 cm3), a long interval time from previous surgical resection and a preoperative KPS higher than 70 [4, 5]. Radiation therapy (RT) has been also proven to be useful in recurrent glioblastoma. However, radiation oncologists have been highly reluctant to re-irradiate local recurrences in the brain in relatively short interval. The assumption that the central nervous system (CNS) tissues are not able to repair radiation injury, limited the use of this local approach, although some increasing evidence exist of the use of radiotherapy in GBM retreatment [12]. These evidences, along with the improvement of neuro-imaging, and the availability of modern high-precision radiotherapy techniques [8–11], allowed a re-evaluation of RT in the clinical practice. In addition, in the recent years, an increased number of clinical trials tested in patients with glioblastoma recurrences, the efficacy of single and/or combined chemotherapy agents [13–21] as well as the benefit of anti-angiogenetic drugs, such as bevacizumab, alone or associated with chemotherapy [22–25] with encouraging results. In any case, even in lack of a standard of care, chemotherapy remains an important treatment option in recurrent GBM. This is mainly due to the fact that a considerable number of recurrences develop as diffuse infiltrating masses, sometime involving also multiple distant sites. Indeed, these patients are those in which the clinical performance is usually deteriorating. Some recurrences, instead, develop around the previous tumor site as defined masses. In this particular setting of patients, the combined use of various treatment approaches might be beneficial. Based on these observations, the aim of the study was to evaluate the survival benefits and toxicity profile of systemic chemotherapy in recurrent brain glioma with or without local therapy. Results were evaluated in terms of toxicity, rate of progression free survival (PFS) and patients overall survival (OS).

## Methods

The present retrospective study includes patients with a MRI evidence of recurrent or progressive GBM, occurring at least 3 months after the end of RT, in order to exclude pseudo-progression. At the initial diagnosis, all patients had undergone open-surgery resection, followed by radiotherapy with concomitant and adjuvant temozolomide according to the Stupp scheme [1]. At the time of recurrence, they were evaluated for salvage treatment. From January 2006 to April 2014, 43 consecutive patients were included in this retrospective study. Twenty-two (51 %) were male and 21 (49 %) female with a median age of 51 years (range 27–80 years). All patients were treated in agreement with the Helsinki declaration. This study is a summary of a retrospective analysis to the treatment charts. The Humanitas Institute’s ethical committee does not require a formal approval in case of retrospective study in which a formal consent for handling patient medical data was obtained at the time of admission according to the deliberation of the national agency for clinical studies of 2008.

Patients characteristics and treatments at diagnosis are shown in Table 1. Two groups of patients were analyzed. Twenty-one (49 %) patients underwent combined treatment, surgery and/or stereotactic radiation therapy plus chemotherapy. Twenty-two (51 %) received chemotherapy alone. The two groups were balanced in terms of patients characteristics and disease status as detailed in Table 2. Inclusion criteria for both groups are: outpatients with KPS greater than 70, an interval time from previous surgery or radiotherapy longer than 6 months and no multifocal disease.

**Table 1:** Patients characteristics and treatments at diagnosis

	No. (%)
Sex	
Female	21 (49)
Male	22 (51)
Median age years (range)	51 (27–80)
MGMT promoter methylation status	
Methylated	18 (42)
Unmethylated	8 (19)
Unknown	17 (39)
IDH mutation	
Present	6 (14)
Absent	20 (47)
Unknown	17 (39)
KPS	
100	11 (26)
90	19 (44)
80	2 (4)
70	11 (26)
Time to Relapse from initial diagnosis	
≤ 12 months	21 (49)
> 12–24 months	13 (30)
> 24 months	9 (21)
Treatment at Initial Diagnosis	
Surgery	43 (100)
Complete Resection (CR)	24 (56)
Subtotal Resection (SR)	5 (12)
Partial Resection (PR)	12 (28)
Biopsy	2 (4)
Radiotherapy	43 (100)
CT Concomitant and adjuvant (TMZ)	43 (100)

*MGMT* methylguanine-DNA methyltransferase, *IDH* isocitrate dehydrogenase, *KPS* karnofsky performance status, *TMZ* Temozolomide

**Table 2:** Characteristics of patients in relation to different therapeutic approaches: combined treatment (chemotherapy CT, Surgery and Radiotherapy RT) versus chemotherapy only according to gender, age, KPS , MGMT promoter status, IDH mutation, time between initial diagnosis and recurrence and recurrent tumor volume

Factors	CT + surgery and/or RT n. pts 21 (49 %)	CT only n. pts 22 (51 %)	*p* value
Gender			
Female	10 (49)	11 (50)	0.9
Male	11 (51)	11 (50)	
Median age	50 years (range 27–75 years)	53 years (range 38–80 year)	0.4
MGMT promoter status			
Methylated	9	9	0.9
Unmethylated	4	4	
Unknown	8	9	
IDH mutation			
Present	5	1	0.17
Absent	8	12	
Unknown	8	9	
KPS			
100	11	0	<0.01
90	5	14	
80	2	0	
70	3	8	
Time to Relapse from initial diagnosis			
≤ 12 months	6	15	<0.01
> 12–24 months	7	6	
> 24 months	8	1	
Median volume of recurrent disease (cm^3^)			
< 35 cm^3^	10	10	0.7
≥ 35 cm^3^	11	12	

*MGMT* methylguanine-DNA methyltransferase, *IDH* Isocitrate dehydrogenase

Surgery consisted in subtotal resection (SR) for all patients [26]. For radiation therapy, to precisely define the exact extension of tumor, CT scans, enhanced T1-MRI, FLAIR-MRI sequences and [11C]MET-PET were used. Automatic rigid co-registration eventually manually corrected was performed. The total dose prescribed was 25 Gy in 5 fractions. The hypofractionated approach was chosen to improve logistic issues, patient compliance and provide a more aggressive radiation treatment. Plans were processed using intensity modulated therapy by means of volumetric modulated arc therapy in its RapidArc form (Varian medical system, Palo Alto - USA) to ensure maximal dose conformity and rapid dose falloff toward critical structures. Patients were treated with 6MV photon beams generated by Varian linear accelerators. In both groups second line chemotherapy was used and consisted of Fotemustine (75–100 mg/m^2^), re-challenge TMZ (50–100 mg/m^2^) and dose dense TMZ (100 mg/m^2^) as 1 week off/1 week on scheme. TMZ was administered to patients who demonstrated response to it during the treatment at diagnoses.

### Outcome evaluation

Clinical outcome was evaluated by clinical neurological examination and brain MRI, 1 month after treatment and then every 3 months at follow up. Response was recorded using the Response Assessment in Neuro-Oncology (RANO) criteria [27]. Hematologic and non-hematologic toxicities were graded according to Common Terminology Criteria for Adverse Events version 4.0.

### Statistical analysis

Standard descriptive statistics (mean standard deviation and cross tabulation analysis) was used to describe the data general behavior. Survival and recurrence time observations were plotted according to the method of Kaplan and Meier, and were starting from the date of recurrence. Univariate analysis was performed with the log-rank test to investigate the prognostic role of individual variables. Backward stepwise multivariate Cox regression model was used as a method to estimate the independent association of a variable set with overall/progression free survival. The model is performed in a backward stepwise fashion with a probability to remove-level set to 0.25. All analyses are sex and age adjusted.

## Results

**Fig. 1:**
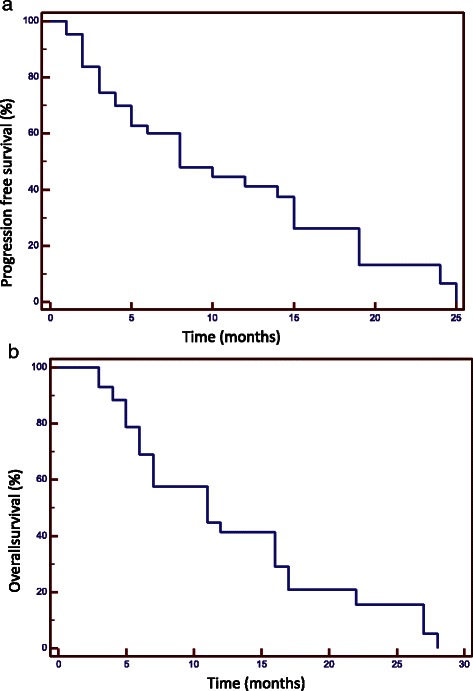
Progression free survival (**a**) and Overall Survival (**b**)

**Fig. 2:**
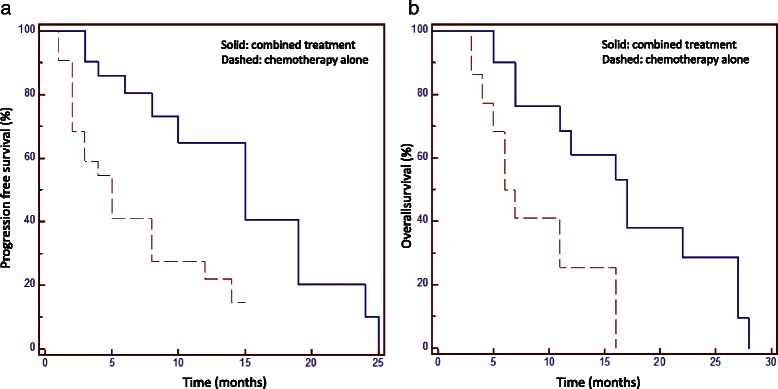
Progression free survival (**a**) and Overall Survival (**b**) in relation with different treatment modalities. Solid line: combined treatment; dashed line: radiotherapy alone (RT)

The median interval time from the initial diagnosis to the recurrence was 13 months (range 6–78 months) and all the 43 patients received further treatments. Hematologic toxicity G1-2 was recorded in 8 patients (20 %). In no patients a delay of drug administration was required. No severe acute hematologic toxicity requiring interruption of the chemotherapy administration was recorded. Similarly, no acute worsening of neurologic state occurred during treatment, and neurological examination scores remained stable. During follow up, an increase of antiepileptic drugs (AEDs) and corticosteroids administration was needed in the case of disease progression. At the same, asymptomatic radio-necrosis (G1-G2) was observed in 5 patients. No G3-G4 radio-necrosis was recorded and none patients needed re-surgery All patients were treated with 25Gy in 5 fractions with plan normalization at mean target dose so that uniform prescription was obtained for all patients. Dose to organs at risk was kept below tolerance levels for all patients (these were near-to-maximum dose <12Gy for brain stem, <8Gy for optic structures, <12Gy for cochlea). The median follow up time from recurrence was 7 months (range 3–28 months). At the time of last observation 12 patients (28 %) were alive and 31 (72 %) were dead. The 1-year PFS was 41 %; the 1-year OS was 42 %. The median PFS and OS from recurrence were 8 months (range 3–28 months; SD 7.4) and 11 months (range 3–38 months; SD 7.5) respectively. No prognostic factors (including age, sex, performance status and recurrence volume as well as IDH1 mutation and MGMT promoter status) resulted significant to univariate analysis with exception of the different modality treatment used; results are summarized in Table 3. Considering the different treatment approaches, patients submitted to combined treatment had the best outcome. The 1-year PFS was 65 % for combined treatment; it resulted 22 % for chemotherapy alone (*p* < 0.01; HR 2.5; CI95 % 1.21–5.28). The median PFS was 15 and 5 months respectively. The 1-year OS was 69 % for the combined approach and 26 % for chemotherapy alone (*p* < 0.01; HR 2.6; CI95 % 1.24–5.45). The median OS was 17 and 6 months respectively. Figures 1 and 2 show the various survival actuarial curves.

**Table 3:** Overall Survival (OS) and Progression free survival (PFS). Univariate regression

	*p*-value (OS)	*p*-value (PFS)
Sex	0.24	0.23
Age	0.29	0.31
EOR at diagnosis	0.511	0.42
MGMT	0.14	0.11
Target Volume	0.87	0.96
IDH1	0.37	0.48
KPS	0.38	0.32
Treatment	0.01	0.004

*KPS* karnofsky performancs status, *IDH* isocitrate dehydrogenase, *MGMT* methylguanine-DNA methyltransferase, *EOR* extension of resection

## Discussion

Available treatments for recurrent GBM include chemotherapy, RT, surgery, and of course, best supportive care. In case of recurrence, the best treatment option is not yet defined, and it is a matter of large debate.

To date, chemotherapy alone remains the treatment of choice in recurrent GBM. Various anti-neoplastic agents have been tested. Several dosing schedules of TMZ alone or other agents combined was associated with a PFS at 6 months of 45 % and a 1 year OS of 20 % [14, 28–30]. In addition to TMZ based regimens, nitrosourea-based regimens employing carmustine (BCNU) monotherapy or lomustine (CCNU) combined with procarbazine and vincristine (PCV) demonstrated similar results in terms of survival at the cost of a greater hematologic toxicity [31–33]. Some studies [22] evaluated the efficacy of different chemotherapy regimens, e.g. bevacizumab alone or in combination with irinotecan, showing some synergistic effect. In the present study, more than half of the patients (55 %) received chemotherapy alone. The results observed are comparable with those of literature with a 1-year PFS and 1-YEAR OS of 22 and 2 % respectively. Details about some published studies are summarized in Table 4. If chemotherapy alone is the main treatment used, the results in term of disease control and survival are still unsatisfactory; the addition of local treatments could be a valuable approach to improve outcome for recurrent HGG patients. The role of local treatment needs still to be assessed. Surgery is not considered as a standard of cure. In different published retrospective studies, the median OS in patients submitted to second surgery was 6 months on the average [4, 5]. In a recent study evaluating the effect, frequency, and complications of repeated surgical resection and value of additional adjuvant therapy, it was highlighted that patients receiving CT or Radiosurgery (SRS) had a significantly prolonged survival compared to those undergoing surgery only. The median OS was 8.5 months compared to 3 months in patients treated with surgery alone. The authors concluded that re-surgery may be beneficial only if additional adjuvant treatment options could be further employed [34].

**Table 4:** Main published studies about patient with recurrent high grade glioma (III–IV) treated with chemotherapy alone

Authors	Study	N. PTS	Treatment	PFS (months)	PFS 6 %	OS (months)
Van Den Bent et al. [15]	Phase II rand	108	BCNU or TMZ vs Erlotinib	2.4	24.1	7.3
		54		1.8	11.4	7.7
Brandes et al. [33]	Phase II	40	BCNU	NA	17.5	7.5
Addeo et al. [18]	Phase II	40	Fotemustine	6.7	61	11.1
Brada et al. [14]	Phase II	126	TMZ	2.1	18	5.4
Wich et al. [29]	Phase II	64	TMZ 1wk on/1 wk off	5.5	43.8	NA
Brada et al. [21]	Phase II rand	87	TMZ 5 days	5	NA	8.5
		81	TMZ 21 days	4.2		6.6
		162	PCV	3.6		6.7

*TMZ* temozolomide, *NA* not available, *mos* months, *PFS6* progression free at 6 months

More recently, the use of radiation therapy in case of GBM recurrence has been revisited, evaluating the effect of different modalities of dose delivery such as radiosurgery (SRS), fractionated stereotactic radiotherapy (FSRT) or hypo-fractionated stereotactic radiotherapy (HSRT). In this context, most authors recommend an interval of at least 6 months between the first and the second irradiation [35–37], in order to allow the repair of the radiation damage [12, 38]. The effect of the use of re-irradiation alone are interesting and comparable to those of other single treatment modality, with a median survival of 9 months and acceptable toxicity rates [39] To date, the largest trial was performed by Fokas [36] on 53 GBM patients who were re-irradiated using hypo-fractionated stereotactic radiotherapy (HSRT). Re-irradiation was well tolerated (no acute or late toxicity > grade 2), despite the relatively large median tumor volumes (35.01 ml); the median survival was 9 months, and the 1-year progression-free survival (PFS) was 22 %. Recently, the role of concomitant chemo-radiotherapy in recurrent setting has been also evaluated [40–43] Combs analyzed the toxicity of TMZ combined with FSRT on 6 patients undergone previous RT with TMZ. They showed treatment feasibility maintaining low toxicity without differences between TMZ-naïve and pre-exposed patients. PFS at 6 months was 48 %, higher than most reported data in the literature about HGGs re-treatments [40]. Minniti [41] reported a series of patients with recurrent GBM who received FSRT plus concomitant TMZ. Median OS and PFS were 9.7 and 5 months, respectively. Six- and 12-month OS rates were 84 and 33 %, respectively, and the respective PFS rates were 42 and 8 %.

Arcicasa [42] reported a series of 24 patients, treated with surgery, radiotherapy and chemotherapy using CCNU. Median interval between RT courses was 14 months (range 6–73). All patients received a complete course of RT, and 22 of 24 patients received at least one course of CCNU. Objective responses were seen in 14 evaluable patients: 3 with partial response, 5 with stable disease, and 6 with progressive disease. Median time to progression and overall survival from the onset of retreatment were 8.4 months (range 1–22) and 13.7 months (range 1–63+), respectively. Glass [43] showed the feasibility of a combined treatment consisting of fractionated stereotactic radiotherapy (SRT) with cis-platinum (CDDP),with a median response duration of 4.6 months and median survival of 13.7 months.

In the present study. 21 patients received chemotherapy plus local treatments (surgery and/or radiation therapy). The median time to progression was 15 months sand 1-year PFS was 65 %; the median and 1-year OS was 17 months and 69 % respectively. These data improves what reported in literature for HGG re-treatments. To our knowledge, no studies comparing the effect of multimodality treatments in GBM recurrence has been published at now. As previously stated, the main aim of this study was to evaluate the efficacy of combined treatment compared with chemotherapy alone. Specifically, we wished to verify if the combination of local treatments, surgery and/or radiation therapy, to a systemic treatment may improve the outcome of these patients. We observed a median OS of 17 months versus 6 months and about 30 % of patients alive at 2 years versus 0 %. The addition of surgery and/or RT is not burdened by an increase of severe toxicity and no peri-operative mortality occurred. This suggest, that the addition of local treatment may be beneficial in a particular setting of patients. Only patients with recurrences limited to the site of the previous primary site and appearing as a well defined mass, with a good performance status, and an interval time between previous surgery and/or RT longer than 6 months were included in this analysis. Besides this, the characteristics of the two groups were comparable. The main limitations of the present study are the retrospective nature and the low number of patients. Our data did not show and differences in relation to age, KPS, IDH1 mutation and MGMT promoter status, in our series did not modify survival. Probably, these results are related to the small sample size of the study.

## Conclusion

This study suggest that in case of GBM recurrences, the use of local treatment (surgery and/or radiotherapy) achieves better results when compared with chemotherapy. In this setting, the combined treatment achieves better PFS and OS with minor toxicity. In addition, a multidisciplinary evaluation is recommended to achieve the best choice of treatment schedule for these highly selected patients.

## References

[1] Stupp R, Hegi M, Mason W, van den Bent M, Taphoorn M, Janzer R (2009). Effects of radiotherapy with concomitant and adjuvant temozolomide versus radiotherapy alone on survival in glioblastoma in a randomised phase III study: 5-year analysis of the EORTC-NCIC trial. Lancet Oncol.

[2] National Comprehensive Cancer Network NCCN guidelines: central nervous system cancers. http://www.nccn.org/professionals/physiciangls/fguidelines.asp 2012.

[3] Minniti G, Amelio D, Amichetti M, Salvati M, Muni R, Bozzao A (2010). Patterns of failure and comparison of different target volume delineations in patients with glioblastoma treated with conformal radiotherapy plus concomitant and adjuvant temozolomide. Radiother Oncol.

[4] Barbagallo G, Jenkinson M, Brodbelt A (2008). “Recurrent” glioblastoma multiforme, when should we reoperate?. Br J Neurosurg.

[5] Park J, Hodges T, Arko L, Shen M, Dello Iacono D, McNabb A (2010). Scale to predict survival after surgery for recurrent glioblastoma multiforme. J Clin Oncol.

[6] Wick W, Platten M, Weller M (2009). New (alternative) temozolomide regimens for the treatment of gliomas. Neuro Oncol.

[7] Wick W, Weller M, Weiler M, Matchelor T, Yung A, Platten M (2011). Pathway inhibition: emerging molecular targets for treating glioblastoma. Neuro Oncol.

[8] Combs S, Gutwein S, Thilmann C, Huber P, Debus J, Schulz-Ertner D (2005). Stereotactically guided fractionated re-irradiation in recurrent glioblastoma multiforme. J Neurooncol.

[9] Combs S, Widmer V, Thilmann C, Hof H, Debus J, Schulz-Ertner D (2005). Stereotactic Radiosurgery (SRS) – treatment option for recurrent Glioblastoma Multiforme (GBM). Cancer.

[10] Biswas T, Okunieff P, Schell M, Smudzin T, Pilcher W, Bakos R (2009). Stereotactic radiosurgery for glioblastoma: retrospective analysis. Radiat Oncol.

[11] Fuller C, Choi M, Forthuber B, Wang S, Rajagiriyil N, Salter B (2007). Standard fractionation Intensity Modulated Radiation Therapy (IMRT) of primary, recurrent glioblastoma multiforme. Radiat Oncol.

[12] Niyazi M, Jansen N, Rottler M, Ganswindt U, Belka C (2014). Recurrence pattern analysis after re-irradiation with bevacizumab in recurrent malignant glioma patients. Radiat Oncol.

[13] Lamborn K, Yung W, Chang S, Wen P, Cloughesy T, DeAngelis L (2008). Progression-free survival: an important end point in evaluating therapy for recurrent high-grade gliomas. Neuro Oncol.

[14] Brada M, Hoang-Xuan K, Rampling R, Dietrich P, Dirix L, Macdonald D (2001). Multicenter phase II trial of temozolomide in patients with glioblastoma multiforme at first relapse. Ann Oncol.

[15] van den Bent M, Brandes A, Rampling R, Kouwenhoven M, Kros J, Carpentier A (2009). Randomized phase II trial of erlotinib versus temozolomide or carmustine in recurrent glioblastoma: EORTC Brain Tumor Group study 26034. J Clin Oncol.

[16] Reithmeier T, Graf E, Piroth T, Trippel M, Pinsker M, Nikkah G (2010). BCNU for recurrent glioblastoma multiforme: efficacy, toxicity and prognostic factors. BMC Cancer.

[17] Balana C, Villa S, Teixidor P (2011). Evolution of care for patients with relapsed glioblastoma. Expert Rev Anticancer Ther.

[18] Addeo R, Caraglia M, De Santi M, Montella L, Abbruzzese A, Parlato C (2011). A new schedule of fotemustine in temozolomide-pretreated patients with relapsing glioblastoma. J Neurooncol.

[19] Khan R, Raizer J, Malkin M, Bazylewicz K, Abrey L (2002). A phase II study of extended low-dose temozolomide in recurrent malignant gliomas. Neuro Oncol.

[20] Norden A, Lesser G, Drappatz J, Ligon K, Hammond S, Lee E (2013). Phase II study of dose-intense temozolomide in recurrent glioblastoma. Neuro Oncol.

[21] Brada M, Stenning S, Gabe R, Thompson L, Levy D, Rampling R (2010). Temozolomide versus procarbazine, lomustine, and vincristine in recurrent high-grade glioma. J Clin Oncol.

[22] Friedman H, Prados M, Wen P, Mikkelsen T, Schiff D, Abrey L (2009). Bevacizumab alone and in combination with irinotecan in recurrent glioblastoma. J Clin Oncol.

[23] Kreisl T, Kim L, Moore K, Duic P, Royce C, Stroud I (2009). Phase II trial of single-agent bevacizumab followed by bevacizumab plus irinotecan at tumor progression in recurrent glioblastoma. J Clin Oncol.

[24] Raizer J, Grimm S, Chamberlain M, Nicholas M, Chandler J, Muro K (2010). A phase 2 trial of single agent bevacizumab given in an every-3-week schedule for patients with recurrent high-grade gliomas. Cancer.

[25] Desjardins A, Reardon D, Coan A, Marcello J, Herndon J, Bailey L (2012). Bevacizumab and daily temozolomide for recurrent glioblastoma. Cancer.

[26] Sanai N, Polley M, McDermott M, Parsa A, Berger M (2011). An extent of resection threshold for newly diagnosed glioblastoma. J Neurosurg.

[27] Wen P, Macdonald D, Reardon D, Cloughesy T, Sorensen A, Galanis E (2010). Updated response assessment criteria for high-grade gliomas: response assessment in neuro-oncology working group. J Clin Oncol.

[28] Wick A, Pascher C, Wick W, Jauch T, Weller M, Bogdahn U (2009). Rechallenge with temozolomide in patients with recurrent gliomas. J Neurol.

[29] Wick A, Felsberg J, Steinbach J, Herrliner U, Platten M, Blaschke B (2007). Efficacy and tolerability of temozolomide in an alternating weekly regimen in patients with recurrent glioma. J Clin Oncol.

[30] Brandes A, Tosoni A, Cavallo G, Bertorelle R, Gioia V, Franceschi E (2006). Temozolomide 3 weeks on and 1 week off as first-line therapy for recurrent glioblastoma: phase II study from Gruppo Italiano Cooperativo di Neuro-Oncologia (GICNO). Br J Cancer.

[31] Weller M, Muller B, Koch R, Mamberg M, Krauseneck P (2003). Neuro-Oncology Working Grp German, C Neuro-oncology working group 01 trial of nimustine plus teniposide versus nimustine plus cytarabine chemotherapy in addition to involved-field radiotherapy in the first-line treatment of malignant glioma. J Clin Oncol.

[32] Brandes A, Tosoni A, Basso U, Reni M, Valduga F, Monfardini S (2004). Second-line chemotherapy with irinotecan plus carmustine in glioblastoma recurrent or progressive after first-line temozolomide chemotherapy: a phase II study of the Gruppo Italiano Cooperativo di Neuro-Oncologia (GICNO). J Clin Oncol.

[33] Brandes A, Tosoni A, Amista P, Nicolardi L, Grosso D, Berti F (2004). How effective is BCNU in recurrent glioblastoma in the modern era? A phase II trial. Neurology.

[34] Mandl E, Dirven C, Buis D, Postma T, Vandertop W (2008). Repeated surgery for glioblastoma multiforme: only in combination with other salvage therapy. Surg Neurol.

[35] Combs S, Debus J, Schulz-Ertner D (2007). Radiotherapeutic alternatives for previously irradiated recurrent gliomas. BMC Cancer.

[36] Fokas E, Wacker U, Gross M, Henzel M, Encheva E, Engenhart-Cabillic R (2009). Hypofractionated stereotactic reirradiation of recurrent glioblastomas: a beneficial treatment option after high-dose radiotherapy?. Strahlenther Onkol.

[37] Ernst-Stecken A, Ganslandt O, Lambrecht U, Sauer R, Grabenbauer G (2007). Survival and quality of life after hypofractionated stereotactic radiotherapy for recurrent malignant glioma. J Neurooncol.

[38] Lawrence Y, Li X, el Naqa I, Hahn C, Marks L, Merchant T (2010). Radiation dose–volume effects in the brain. Int J Radiat Oncol Biol Phys.

[39] Henke G, Paulsen F, Steinbach JP, Ganswindt U, Isijanov H, Kortmann R (2009). Hypofractionated reirradiation for recurrent malignant glioma. Strahlenther Onkol.

[40] Combs S, Bischof M, Welzel T, Hof H, Oertel S, Debus J (2008). Radiochemotherapy with temozolomide as re-irradiation using high-precision fractionated stereotactic radiotherapy (FSRT) in patients with recurrent glioma. J Neurooncol.

[41] Minniti G, Armosini V, Salvati M, Lanzetta G, Caporello P, Mei M (2011). Fractionated stereotactic reirradiation and concurrent temozolomide in patients with recurrent glioblastoma. J Neurooncol.

[42] Arcicasa M, Roncadin M, Bidoli E, Dedkov A, Gigante M, Trovo M (1999). Reirradiation and lomustine in patients with relapsed high-grade gliomas. Int J Radiat Oncol Biol Phys.

[43] Glass J, Silverman CL, Axelrod R, Corn B, Andrews D (1997). Fractionated stereotactic radiotherapy with cis-platinum radiosensitization in the treatment of recurrent, progressive, or persistent malignant astrocytoma. Am J Clin Oncol.

